# Identified Medications Causing Medication-Related Osteonecrosis of the Jaw: A Literature Review

**DOI:** 10.7759/cureus.86645

**Published:** 2025-06-24

**Authors:** Nasimeh Baghalipour, Omid Moztarzadeh, Christos Micopulos, Walla Samara, Lukas Hauer

**Affiliations:** 1 Department of Stomatology, Faculty of Medicine in Pilsen, Charles University, University Hospital Pilsen, Pilsen, CZE; 2 Department of Anatomy, Faculty of Medicine in Pilsen, Charles University, University Hospital Pilsen, Pilsen, CZE

**Keywords:** antiangiogenic drugs, antiresorptive agents, drug-induced osteonecrosis, medication-related osteonecrosis of the jaw (mronj), mronj prevention

## Abstract

Medication-related osteonecrosis of the jaw (MRONJ) is a serious adverse event that occurs in patients treated with certain medications, leading to a major side effect that can result in substantial morbidity. MRONJ is known to be associated with antiresorptive (AR) and antiangiogenic (AA) medicines, but recent evidence suggests that other medications may also be involved. Recognizing a broad range of drug-induced risk factors is important, notably in light of the increasing number of drugs associated with MRONJ. The primary objective of this review is to update and synthesize findings from the past 22 years (2003 to April 2025) regarding medications associated with MRONJ, expanding beyond the traditional AR and AA drugs to include more recent and developing therapeutic classes. This review combines findings from recent literature, including studies on AR and AA medications, as well as other drug classes, such as targeted cancer therapies and immunomodulatory agents. This review provides updated insights into MRONJ for healthcare practitioners and dentists, emphasizing the significance of risk assessment, early recognition, and multidisciplinary management to improve patient care and outcomes. Given its potential to cause significant morbidity and complicate dental and medical treatments, MRONJ presents a critical concern in clinical practice, underscoring the need for heightened awareness among healthcare professionals.

## Introduction and background

Medication-related osteonecrosis of the jaw (MRONJ) is defined by the American Association of Oral and Maxillofacial Surgeons (AAOMS) as exposed bone or bone that can be probed through an intraoral or extraoral fistula in the maxillofacial area, persisting for more than eight weeks in patients with current or prior exposure to medications known to cause the condition, without a history of radiation therapy or metastasis to the jaw [1]. MRONJ has become an increasingly recognized complication, particularly among patients receiving pharmacologic treatments for bone-related conditions. It is most commonly associated with the use of antiresorptive (AR) agents, such as bisphosphonates (BPs) and denosumab, which are frequently prescribed to treat conditions like osteoporosis, bone issues related to malignancy, and Paget’s disease of bone. Although these medications prevent bone resorption, long-term use of them can disrupt natural bone remodeling and result in MRONJ [1]. In addition to denosumab and BPs, which remain the most recognized contributors to MRONJ, new research indicates that antiangiogenics (AAs), corticosteroids, and immunomodulatory medications may potentially play a role in the pathophysiology of MRONJ [2,3]. This has led to a rising awareness that MRONJ is not only associated with AR and AA therapy but could also involve a wider range of pharmacologic agents, especially in patients undergoing polypharmacy [4].

The purpose of this review is to enhance awareness and promote better clinical decision-making in managing patients at risk of MRONJ and also to provide a current synthesis of the pharmacological compounds linked to MRONJ, with an emphasis on the recently discovered drug classes involved in its development. By analyzing these drugs' mechanisms of action, we aim to provide a clearer understanding of how they contribute to MRONJ, as well as the associated risk factors. However, this review evaluates both traditional and novel drug-related causes of MRONJ.

## Review

Methodology

Study Design

This review was conducted as a narrative literature review to identify and evaluate medications associated with MRONJ, including both well-established and emerging pharmacological classes. Although it does not follow a registered systematic protocol, the study was designed with structured methods to ensure transparency and rigor in literature selection and synthesis.

Search Strategy

A structured search of the literature was carried out using PubMed and Google Scholar to retrieve English-language articles published up to April 2025. The search strategy incorporated a combination of keywords and Medical Subject Headings (MeSH) terms such as “medication-related osteonecrosis of the jaw”, “MRONJ”, “antiresorptive”, “antiangiogenic”, “corticosteroids”, “chemotherapy”, “targeted therapy”, and “immunomodulators”. Boolean operators (AND, OR) were used to optimize the precision and breadth of the search. Filters were applied to prioritize clinical trials, observational studies, meta-analyses, expert consensus documents, and relevant case reports describing MRONJ in association with pharmacological agents beyond the traditional scope.

Additional exploratory searches were performed in the Cochrane Library, Web of Science, and Scopus databases; however, these searches did not yield additional medications associated with MRONJ beyond those already included in our review. We acknowledge that the search scope and database familiarity may limit the comprehensiveness of this review.

Eligibility Criteria

Studies were included if they addressed medications linked to the development of MRONJ, presented clinical cases or cohorts involving drug-associated MRONJ, examined the pathophysiological mechanisms of MRONJ in relation to pharmacologic agents, or offered management and prevention strategies. Articles were excluded if they were non-English or lacked clinical relevance to drug-induced MRONJ.

Study Selection

**Figure 1:**
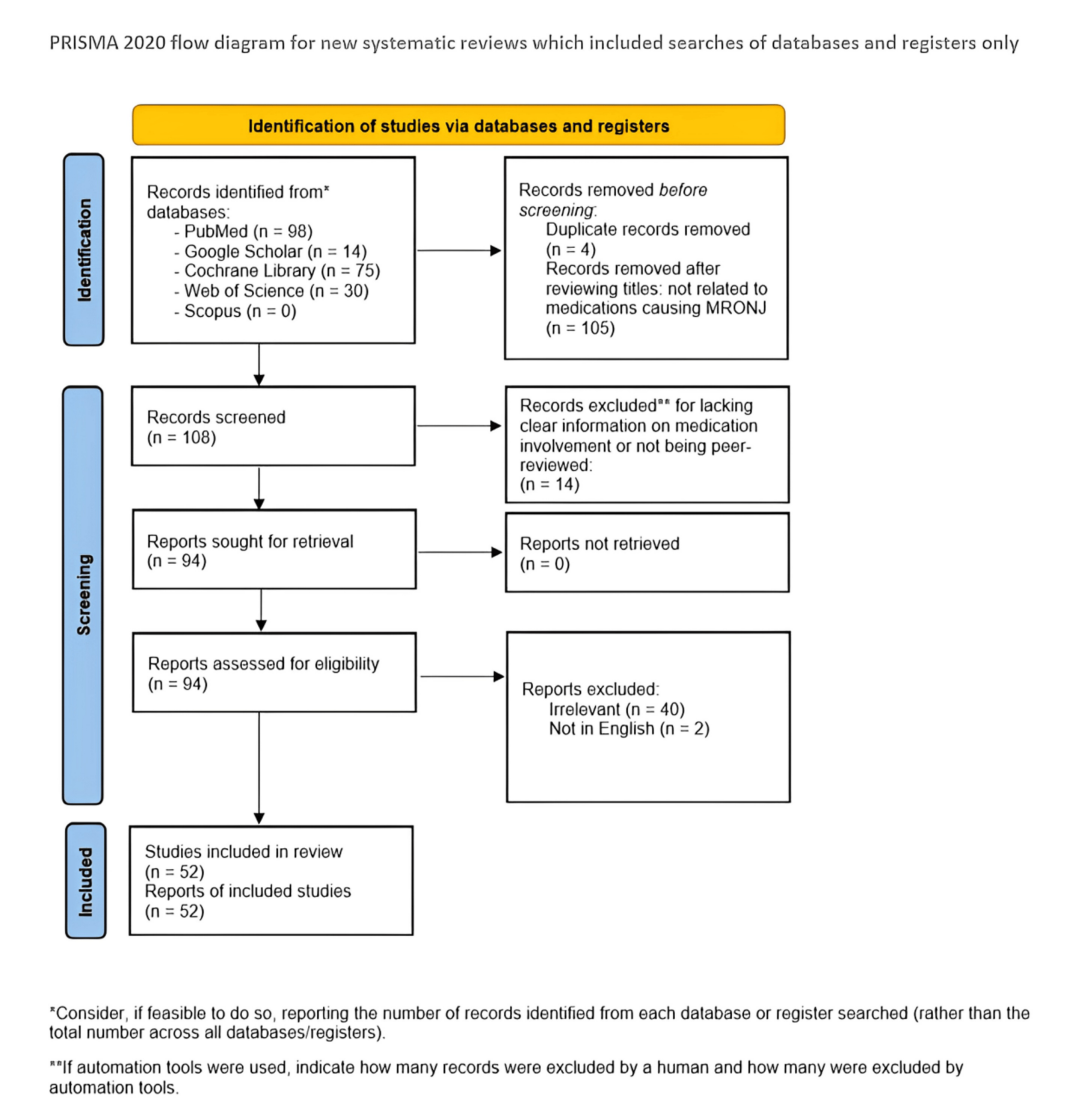
PRISMA 2020 flow diagram of the study selection process PRISMA: Preferred Reporting Items for Systematic reviews and Meta-Analyses Image source: Page et al. (2021) [5]; Creative Commons Attribution (CC BY) license

The selection process followed the Preferred Reporting Items for Systematic reviews and Meta-Analyses (PRISMA) 2020 guidelines [5]. A total of 217 records were initially identified through database searches. After removing 4 duplicate records and excluding 105 articles based on title irrelevance during preliminary screening, 108 records remained. These were then screened by title and abstract to assess relevance to the inclusion criteria. Of these, 14 were excluded for failing to meet the eligibility criteria, and 94 full-text articles were assessed for eligibility. Following the application of inclusion and exclusion criteria, 42 articles were excluded. Ultimately, 52 studies were included in the final analysis. The study selection process is illustrated in the PRISMA flow diagram (Figure 1).

Data Extraction and Synthesis

Data extracted from the included studies encompassed medication class, pharmacologic mechanism, patient characteristics, and MRONJ-related risk factors. Given the heterogeneity across study designs and outcomes, the findings were synthesized narratively to highlight patterns and themes relevant to MRONJ development and clinical management.

Results

This review synthesized findings from a wide range of published literature, including primary research articles and case reports. As a narrative literature review, the emphasis was placed on thematically analyzing systemic and pharmacological risk factors associated with MRONJ rather than tabulating individual study characteristics. While a formal risk of bias assessment was not performed, only peer-reviewed, clinically relevant literature was included to ensure the reliability and relevance of the findings.

The reviewed literature revealed a diverse range of systemic risk factors contributing to MRONJ, particularly among patients treated with both conventional and emerging pharmacological agents. Detailed findings are organized into thematic sections, including systemic risk factors, traditional and novel medications associated with MRONJ, and case reports.

Systemic Risk Factors Associated With MRONJ

Chemotherapy for malignancies such as cancers of the breast, prostate, lung, renal, and colon; multiple myeloma (MM); as well as corticosteroid use, diabetes, tobacco use, and cardiovascular diseases (e.g., angina and hypertension) are established systemic risk factors for MRONJ. Additionally, oncological patients receiving intravenous BPs or high-dose denosumab are at an increased risk. Other systemic factors include osteoporosis, rheumatoid arthritis (RA), Sjögren’s syndrome, sarcoidosis, hypocalcemia, osteomalacia, Paget’s disease of bone, vitamin D deficiency, erythropoietin therapy, cyclophosphamide therapy, obesity, and alcohol intake, which also elevate the risk of MRONJ [6].

Moreover, angiogenesis inhibitors, tyrosine kinase inhibitors (TKIs), monoclonal antibodies (mAbs), mammalian target of rapamycin (mTOR) inhibitors, and immunosuppressant comorbidities have been reported as possible risk factors for the development of MRONJ [6]. These systemic conditions and their treatments may compromise bone metabolism and healing, making patients more susceptible to MRONJ when exposed to high-risk medications [7].

Medications Associated With MRONJ

A wide range of medications has been implicated in the development of MRONJ. To provide a structured overview, the agents discussed in this review are categorized and summarized in Table 1, including traditional therapies as well as newer medications associated with this condition, based on current literature and guidelines.

**Table 1:** Medications associated with MRONJ AA, antiangiogenics; AR, antiresorptive; BP, bisphosphonate; mAb, monoclonal antibody; MM, multiple myeloma; MRONJ, medication-related osteonecrosis of the jaw; mTOR, mammalian target of rapamycin; PDGF, platelet-derived growth factor; RTX, rituximab; TKI, tyrosine kinase inhibitor; TNF-α, tumor necrosis factor-alpha; VEGF, vascular endothelial growth factor; VEGFR, vascular endothelial growth factor receptor Data compiled from studies referenced in this review [8-40] Table credit: Nasimeh Baghalipour

Category	Medications/examples	Mechanism of action/risk factors
AR drugs	BPs: alendronate, risedronate, ibandronate, pamidronate, zoledronate, etidronate, clodronate, and tiludronate	Inhibit osteoclast-mediated bone resorption, leading to apoptosis
	Denosumab: Prolia and Xgeva	Reduces bone turnover, interfering with osteoclast formation and function
AA agents	AA drugs targeting VEGF/VEGFR interactions: bevacizumab, aflibercept, and ranibizumab	Inhibit angiogenesis by disrupting VEGF, PDGF, or receptor tyrosine kinase activity, and impair vascularization necessary for bone healing
	AA drugs targeting VEGFR tyrosine kinase: sorafenib, sunitinib, pazopanib, axitinib, cabozantinib, and regorafenib	
	mTOR inhibitors: everolimus, temsirolimus, and sirolimus	
Polypharmacy	Multiple drugs: antiresorptive (BPs and denosumab) + non-AR agents (angiogenesis inhibitors, TKIs, chemotherapy, and corticosteroids)	Combined effects impair bone healing by affecting cytokines, growth factors, and vascularization
Chemotherapeutic agents	Cytotoxic drugs: methotrexate and azacitidine	Impair bone metabolism, vascularization, and immune function, potentially increasing MRONJ risk, especially when combined with newer anti-cancer drugs
Immune modulators and other drugs from case reports	TNF-α inhibitors: infliximab, adalimumab, and etanercept	Affect bone remodeling, osseointegration, and immune response and impair healing
	IL-6 inhibitors: tocilizumab	
	IL-17 inhibitors: secukinumab	
	IL-23 inhibitors: guselkumab	
	Immunomodulatory drugs: thalidomide and bortezomib (used in MM)	
	Additional TKIs: osimertinib and imatinib	
	mAbs used in immunotherapy: RTX, nivolumab, and ipilimumab	
	AXL kinase inhibitor: bemcentinib	
	BRAF inhibitors: dabrafenib and trametinib	
	HER2 inhibitors: trastuzumab and pertuzumab	
	Osteoanabolic agents: romosozumab	
	Hormonal therapies: potentially confounding factor	
	Other immunosuppressant: pomalidomid	

Traditional agents

AR Drugs

**Figure 2:**
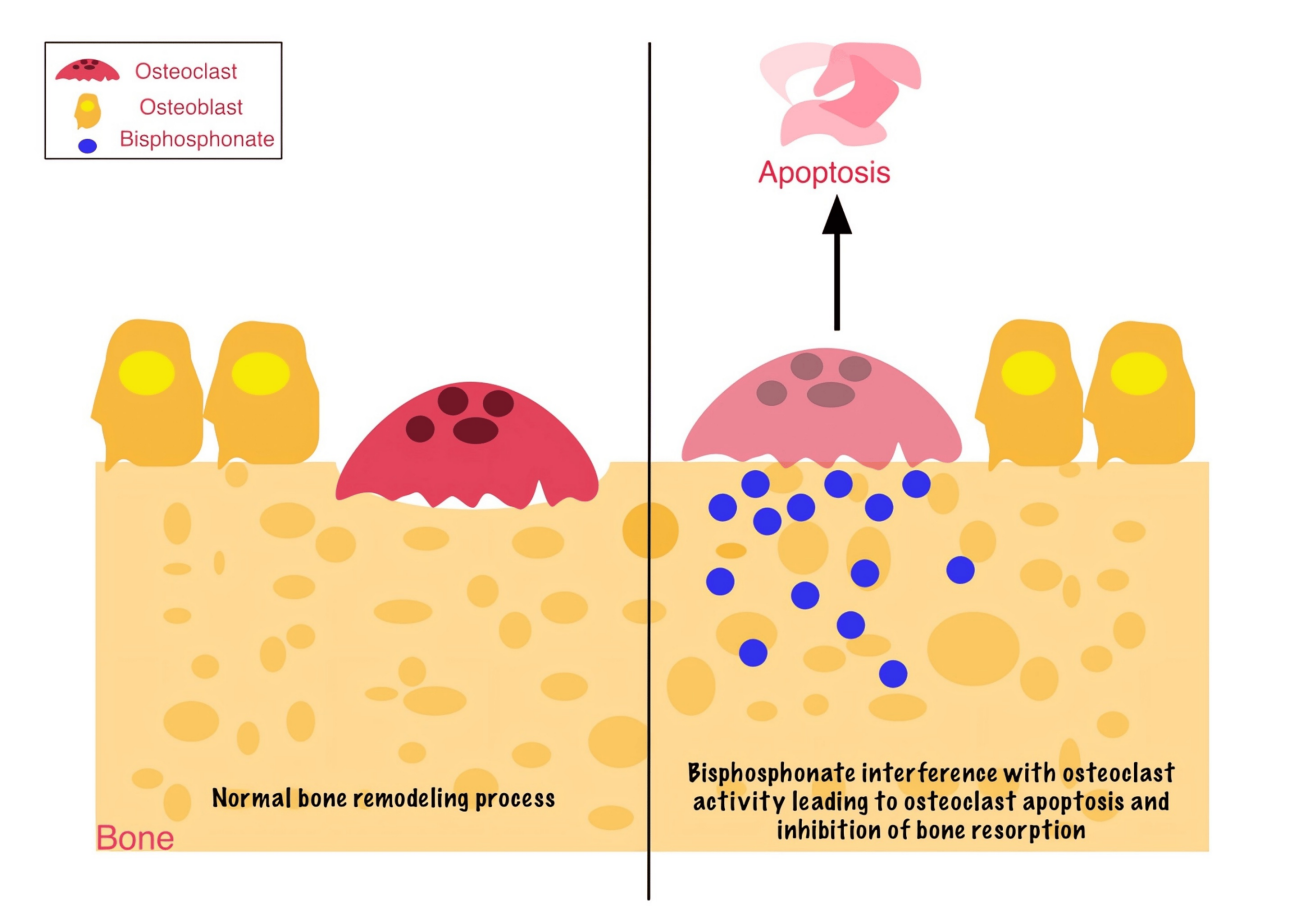
Comparison of normal bone remodeling vs. effects of BPs The left panel shows a balanced cycle of normal bone remodeling, in which osteoblasts promote the formation of new bone while osteoclasts resorb existing bone. The right panel illustrates the impact of BPs, which suppress osteoclast activity and lead to decreased bone resorption and impaired remodeling. Prolonged suppression of osteoclast function can result in excessive bone mineralization, reduced bone turnover, and an increased risk of MRONJ. BP, bisphosphonate; MRONJ, medication-related osteonecrosis of the jaw Image created using Linearity Curve. Credit: Nasimeh Baghalipour. Adapted from [8-10,41]

BPs: BPs are stable inorganic pyrophosphate analogs, which are integrated into bone mineral and subsequently released during bone resorption. Following this, suppressing osteoclast function by leading osteoclasts to undergo apoptosis and inhibiting bone destruction interrupts bone resorption [8,9] (Figure 2). The medication’s effect typically appears within six hours, and its long half-life may last at least 10 years due to its high affinity to bone [10]. Based on their molecular mechanisms of action, BPs are classified into two main groups: non-nitrogen-containing BPs (non-N-BPs) and nitrogen-containing BPs (N-BPs). Non-N-BPs cause apoptosis through metabolizing into non-hydrolysable adenosine triphosphate, which is cytotoxic and stored in osteoclasts, while N-BPs cause osteoclast apoptosis by preventing cholesterol production through blocking farnesyl pyrophosphate synthase. In comparison to non-N-BPs, the nitrogen group dramatically boosts AR effectiveness by up to 10,000 times [2].

BPs have the ability to increase bone density by reducing the bone remodeling process [10] and are therefore widely used to treat skeletal conditions, including (a) osteoporosis; (b) Paget’s disease of bone; (c) malignancy-related conditions like MM, bone metastasis, and hypercalcemia; (d) adjuvant therapy for breast cancer (even without bone metastasis); or (e) other conditions such as osteogenesis imperfecta, synovitis, acne, pustulosis, hyperostosis, and SAPHO syndrome [8]. However, long-term use of BPs has been associated with osteonecrosis of the jaw (ONJ), particularly in cancer patients or those with other risk factors. Furthermore, BPs are known angiogenesis inhibitors, which may affect blood vessel formation [8]. Several BPs, including alendronate, ibandronate, neridronate, pamidronate, risedronate, zoledronate, clodronate, etidronate, and tiludronate, have been implicated in MRONJ due to their inhibition of osteoclast-mediated bone resorption [9].

**Figure 3:**
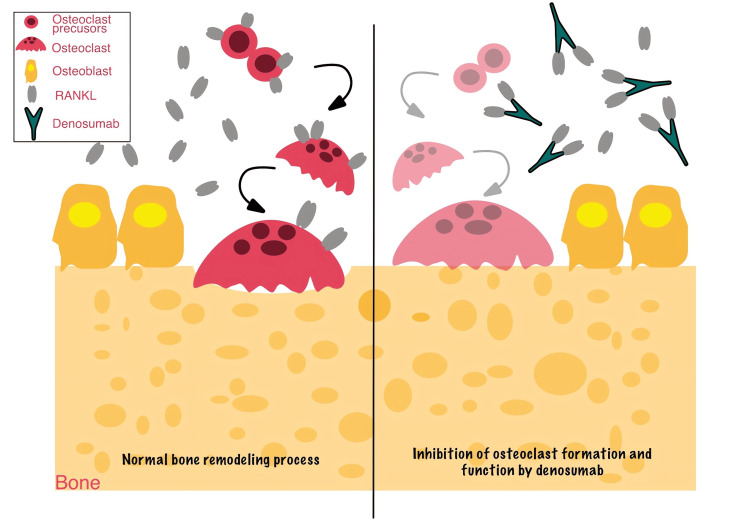
Comparison of normal bone remodeling vs. effects of denosumab The left panel illustrates normal bone remodeling, where osteoclasts resorb bone, and osteoblasts facilitate new bone formation in a balanced cycle. The right panel shows the effect of denosumab, an mAb that inhibits RANKL. By blocking RANKL, denosumab inhibits osteoclast differentiation, activity, and survival, leading to decreased bone resorption. This disruption in bone remodeling can result in excessive bone density, poor healing, and an increased risk of MRONJ. mAb, monoclonal antibody; MRONJ, medication-related osteonecrosis of the jaw; RANKL, receptor activator of nuclear factor kappa-B ligand Image created using Linearity Curve. Credit: Nasimeh Baghalipour. Adapted from [11,12,42]

Denosumab: This medication is an mAb that targets the receptor activator of nuclear factor kappa-B ligand (RANKL). It reduces bone turnover by inhibiting osteoclast formation, survival, and function [11] (Figure 3). This process interferes with osteoclast formation and activity, which may increase the risk of MRONJ, even though it is helpful in treating bone-related disorders [12]. Denosumab has a short half-life, in contrast to BPs, as the RANKL inhibitors cannot bind to the bone. Thus, the effects of denosumab in the bone last shorter than those of BP and generally decrease within six months of the last dose administration [10]. Denosumab is commonly used in two formulations: (a) Prolia, for osteoporosis treatment to reduce hip, vertebral, and non-vertebral fracture risks, and (b) Xgeva, in the treatment of cancer to reduce the risk of skeletal-related events in patients with bone metastases [1].

 *AAs*

**Figure 4:**
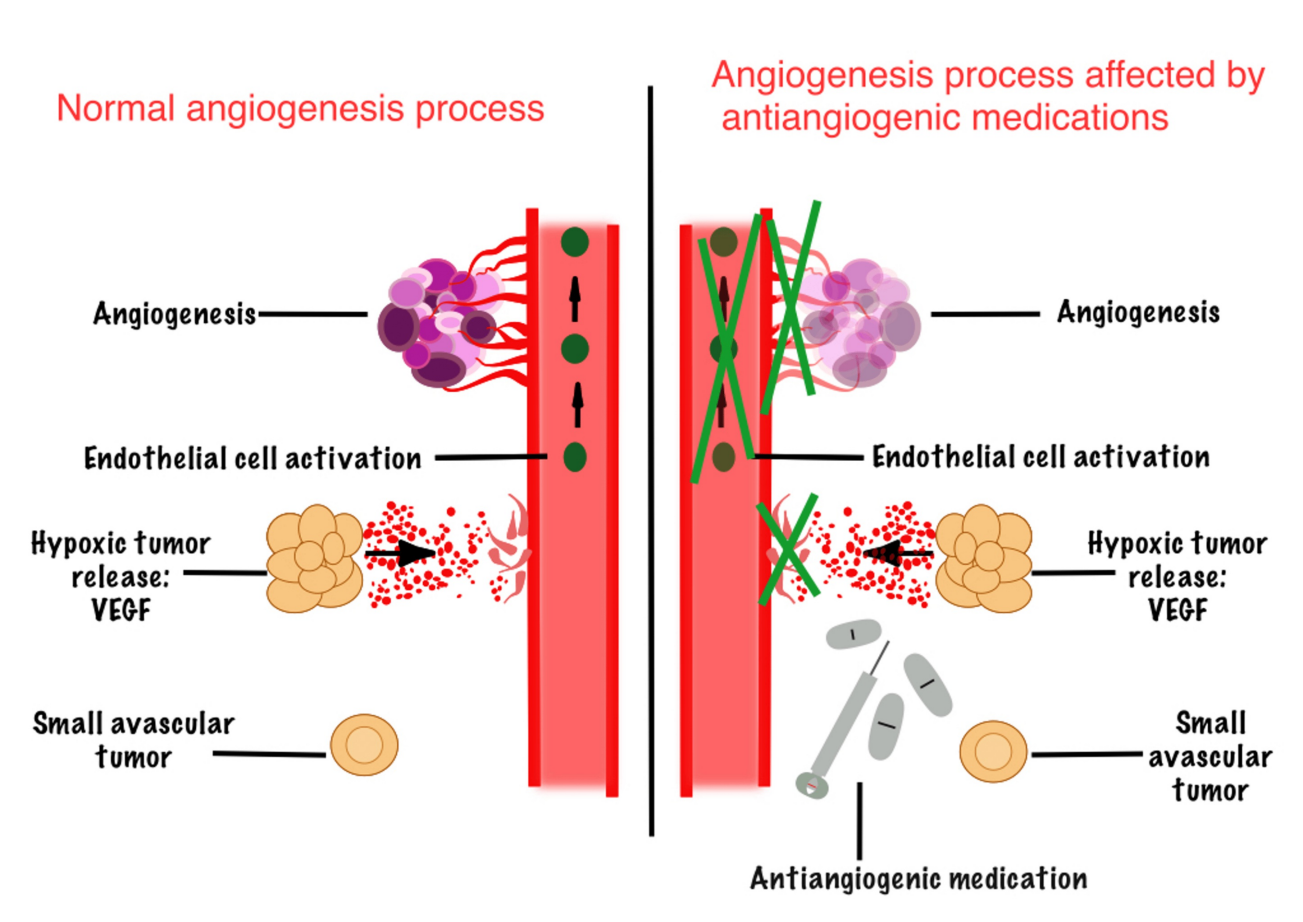
Comparison of normal bone remodeling vs. effects of AA agents The left panel shows normal bone remodeling, where adequate vascularization supports osteoclast and osteoblast function, ensuring balanced bone resorption and formation. The right panel illustrates the effects of AA drugs, which inhibit VEGF and other pathways essential for angiogenesis. Decreased blood vessel formation impairs bone turnover, delays healing, and compromises bone regeneration. This vascular disruption increases susceptibility to MRONJ. AA, antiangiogenics; MRONJ, medication-related osteonecrosis of the jaw; VEGF, vascular endothelial growth factor Image created using Linearity Curve. Image Credit: N. Baghalipour. Adapted from [13,14,15,16,44]

Angiogenesis inhibitor medications are frequently used in the treatment of malignancies, which affect the new blood vessel formation and normal tissue healing process, including the jawbone [13,14] (Figure 4). Bevacizumab, followed by sunitinib, aflibercept, sorafenib, cabozantinib, pazopanib, regorafenib, and dasatinib, disrupts vascularization necessary for bone healing, predisposing patients to MRONJ [43]. TKIs (such as sunitinib), vascular endothelial growth factor (VEGF) inhibitors (such as bevacizumab), and mTOR inhibitors (such as sirolimus, everolimus, and temsirolimus) are the most commonly prescribed medications with a primary AA activity that have a risk of MRONJ [15].

TKIs: They are chemical substances that prevent the kinase activity of receptor tyrosine kinases. The majority are small molecules targeting the intracellular domain of VEGF receptor-2 [15,16]. Due to their AA properties, these medications impair bone healing, thereby increasing the risk of MRONJ [14]. Examples include sunitinib, sorafenib, cabozantinib, and axitinib [17].

Anti-VEGF mAbs: This group of medications, such as bevacizumab and ziv-aflibercept, prevents angiogenesis through binding to VEGF or its receptors [15].

mTOR inhibitors: This type of drug, like everolimus, has AA effects via influencing the synthesis of platelet-derived growth factor (PDGF) and VEGF [15].

Emerging drug associations and immune modulators

Polypharmacy

Cancer patients often use both AR and non-AR treatments at the same time, which increases the risk of several drug interactions. AR drugs, such as denosumab and BPs, prevent skeletal complications and keep the bones from breaking down. Non-AR drugs, such as angiogenesis inhibitors, mTOR inhibitors, TKIs, corticosteroids, and chemotherapy, help control the disease and provide supportive care. However, both types of medications impair wound healing, which is a key contributing factor to MRONJ development. Nevertheless, the severity of complications varies depending on the drug’s dosage, mechanism of action, and route of administration, because these factors impact cytokines and growth factors that are essential for healing [18].

Chemotherapeutic Agents and Hormonal Therapies

Chemotherapy may raise the incidence of MRONJ, particularly when administered in combination with new anti-cancer medications. A study stated that people treated with both chemotherapy and novel anti-cancer medications were at greater risk than those receiving standard chemotherapy alone; however, the exact cause is yet unknown. This elevated risk may be caused by chemotherapy’s immunosuppressive characteristics as well as its cytotoxic effects on bone metabolism and vascularization. Although no definitive conclusions have been drawn, these treatments are considered additional risk factors for MRONJ. Hormonal therapy, however, was not found to be an independent risk factor for MRONJ and is considered a confounding factor in its potential development [19].

Corticosteroids

This type of medication is a significant risk factor for MRONJ, as it impairs bone and soft tissue healing by increasing osteoblast and osteocyte apoptosis. There is limited evidence for direct MRONJ development by this type of medication, despite their association with osteonecrosis in other bones, such as the femur and vertebrae, being reported. To fully understand their role, particularly when combined with other drugs, more research is required [18].

Anti-Tumor Necrosis Factor-Alpha (TNF-α)

TNF-α inhibitors, used for the treatment of inflammatory autoimmune diseases, for instance, in RA, ankylosing spondylitis, Crohn’s disease, inflammatory bowel disease, Behcet’s disease, psoriasis, psoriatic arthritis, and juvenile arthritis, may exacerbate MRONJ. TNF-α and RANKL have similar biological functions, whose inhibition by denosumab is linked to MRONJ. Blocking TNF-α disrupts osteoclast formation, bone remodeling, and immune regulation, impairing healing and increasing infection risk. Research reports instances of MRONJ in patients on TNF-α inhibitors following trauma or dental treatments. Consequently, these inhibitors are to be regarded as a possible risk factor for MRONJ, and throughout treatment with this type of medication, patients need to be closely evaluated and examined for osteonecrotic lesions [20].

Anti-TNF-α drugs for the treatment of inflammatory diseases include infliximab, adalimumab, etanercept, golimumab, and certolizumab pegol.

*Romosozumab* 

This medication is a new mAb for osteoporosis. Romosozumab is the first medication that has both resorption-inhibiting and bone-formation-promoting effects, which have both pro-osteogenic and AR effects since it inhibits sclerostin, a glycoprotein that suppresses bone formation by osteoblasts and enhances bone resorption by osteoclasts. However, it may also induce ONJ, underscoring the potential role of various medications in the development of MRONJ [21].

Case reports and literature evidence

Several case reports illustrate the development of MRONJ in patients exposed to less common drug categories, or MRONJ developed in the absence of BP or denosumab:

IL Inhibitors

Secukinumab, IL-17 inhibitors: Secukinumab is an mAb that blocks the cytokine IL-17A, which is linked to angiogenesis, bone resorption, and inflammation. Secukinumab may disrupt immune response and bone remodeling by inhibiting IL-17A, subsequently raising the risk of MRONJ. It is reported that a 65-year-old female patient with severe psoriasis and psoriatic arthritis, treated with secukinumab since November 2020, was diagnosed with MRONJ despite the absence of AR or AA medications in her medical history. This case report emphasizes the potential risk of MRONJ associated with secukinumab and underscores the importance of monitoring patients on treatment with this drug [22].

Guselkumab, IL-23 inhibitors: A study reports MRONJ diagnosed in a 67-year-old man who has taken guselkumab, an IL-23 inhibitor, to treat psoriatic arthritis and plaque psoriasis. This study found that guselkumab may have a possible association with MRONJ, as the patient has no history of taking AR or AA medications. In summary, the importance of regular follow-up visits is emphasized for patients receiving guselkumab, given the potential side effects associated with immune-modulating medications [23].

Tocilizumab, an IL-6 receptor inhibitor: Tocilizumab is a medication used to treat autoimmune conditions such as RA, and based on a reported case, it may be associated with MRONJ. A patient with severe RA was diagnosed with osteomyelitis in the maxilla and mandible, which progressed to osteonecrosis and was aggravated by tocilizumab. Furthermore, the fact that the patient had no prior history of AR drug exposure is significant since it raises the potential that the immunosuppressive effects of IL-6 suppression could be contributing to poor bone healing and increased susceptibility to MRONJ. Even though there are just a few case reports available currently, more investigation is necessary to fully understand the possible causative relationship between tocilizumab and MRONJ [24].

VEGF Inhibitors

Bevacizumab: A commonly used therapeutic approach to advanced tumors, including metastatic breast cancer, is the inhibition of VEGF-A using mAb agents like bevacizumab. While a case study highlights the probable connection between MRONJ and bevacizumab in a 54-year-old woman who received treatment with bevacizumab without a previous history of BP exposure, smoking, or head and neck radiotherapy, she is diagnosed with MRONJ. However, further randomized studies are needed to develop safer treatment protocols, particularly in patients undergoing oral surgery or dental implants [25].

Aflibercept: Recombinant fusion protein ziv-aflibercept is a VEGF-targeting intravascular agent currently used with FOLFIRI for the treatment of metastatic colorectal cancer. A published case series reported three patients with metastatic gastrointestinal cancer who developed MRONJ following treatment with ziv-aflibercept. This medication has now been implicated in MRONJ, potentially through its inhibition of VEGF, a mechanism similar to that of bevacizumab and sunitinib. Therefore, the authors suggest that patients receiving ziv-aflibercept should be closely monitored, as it is a new medication linked to MRONJ, to detect any potential complications [26].

Ranibizumab: It is a humanized mAb fragment of the IgG kappa isotype, generated through recombinant technology that originates from bevacizumab, containing only its antigen-binding (Fab) region, which, despite its smaller size compared to bevacizumab, gives it a higher affinity for VEGF. This smaller size also makes it particularly suited for intravitreal administration, as it allows for effective diffusion from the vitreous into the retina and choroid, providing therapeutic benefit for neovascular ocular diseases [27]. A case published in the International Journal of Oral and Maxillofacial Surgery described the onset of MRONJ in a patient treated with intravitreal ranibizumab injections for neovascular age-related macular degeneration. Importantly, this patient had no history of BP use, suggesting a potential association between ranibizumab and MRONJ [45].

TNF-α Inhibitors

Infliximab: This medication is a TNF-α inhibitor used to treat various autoimmune disorders, such as Crohn’s disease, which has been recently identified as a possible pharmacological risk factor for developing MRONJ. According to a case report by Oryniak et al. (2024), a 78-year-old man who had received infliximab treatment for ten years suffered from severe bone necrosis in his mandible after having dental extractions. Initially, it was thought that an infectious process due to poor oral hygiene was causing the necrosis, but it was eventually established that MRONJ was mostly caused by the immunosuppressive effects of infliximab medication. This case report highlights the concern that TNF-α may inhibit or impair bone healing and contribute to MRONJ in medically compromised patients, underscoring the need for careful management in such cases [28].

Adalimumab: This medication is recombinant IgG, an mAb that blocks TNF-α by binding to the cytokine itself, preventing its interaction with the protein 55 and protein 75 cell surface receptors, thereby modulating inflammatory and immune responses. Initially approved for RA, it is also used for conditions like Crohn’s disease and psoriasis. This medication can be used alone, but it is often prescribed in combination with methotrexate. While MRONJ is typically associated with BPs and denosumab, a case report described MRONJ development in a 70-year-old RA patient treated with methotrexate and adalimumab, without prior AR or AA therapy. This suggests a potential link between adalimumab and MRONJ, requiring further study [29].

TKI

Osimertinib: This medication is a third-generation epidermal growth factor receptor (EGFR) TKI used for NSCLC with specific EGFR mutations. A case report by Wang et al. (2022) described a 69-year-old woman with non-small-cell lung cancer (NSCLC) who developed MRONJ with a history of four years of osimertinib treatment. This case provides evidence that osimertinib monotherapy may contribute to MRONJ, highlighting the need for monitoring patients on long-term osimertinib therapy for potential MRONJ development, especially in the presence of other risk factors [30].

Imatinib: This medication is a TKI used to treat malignancies like chronic myelogenous leukemia, acute lymphocytic leukemia, and c-kit-positive gastrointestinal stromal tumors. Imatinib suppresses two key regulators of angiogenesis, PDGFR and c-kit, and in some studies, it has been demonstrated that it partially decreases VEGF expression by inhibiting the same factors. Along with its impact on vascular pathways, imatinib also affects bone remodeling by inhibiting c-fms and c-kit signaling, which reduces osteoclast activity and quantity. A case report by Okubo-Sato et al. (2021) describes a rare instance of spontaneous MRONJ in a 52-year-old woman treated with imatinib, without prior AR treatment or radiotherapy in the head and neck area. The case emphasizes that MRONJ can occur even without traditional risk factors, highlighting the need for long-term monitoring and awareness among dental and medical professionals [31]. 

Human EGFR 2 (HER2) Inhibitors

HER2 belongs to the EGFR family and possesses intrinsic tyrosine kinase activity. Upon dimerization, HER2 undergoes autophosphorylation at tyrosine residues in its cytoplasmic domain, triggering downstream signaling cascades that drive cell growth and contribute to tumor development [46]. Two medications, trastuzumab and pertuzumab, are used in the first-line treatment of HER2-positive metastatic breast cancer [32].

Trastuzumab: It is an mAb that blocks the EGFR, and it has previously been identified as an independent risk factor for developing ONJ, specifically in patients who were also taking zoledronic acid [32].

Pertuzumab: This is a drug that inhibits HER2 dimerization and enhances trastuzumab’s effects but has not previously been associated with ONJ [32].

Trastuzumab has AA effects, and these effects become stronger when pertuzumab is added. However, neither drug directly affects osteoclasts [32]. Manzie et al. (2020) report a case of the first documented instance of ONJ in a 76-year-old woman receiving trastuzumab and pertuzumab for HER2-positive metastatic breast cancer, despite no prior use of BPs, denosumab, or other ONJ-associated drugs. The patient was diagnosed with stage II MRONJ, and her case suggests that anti-HER2 therapies suggest the need for increased clinical vigilance, while further research is needed to clarify the incidence and underlying mechanisms [32].

mAbs Used in Immunotherapy

Rituximab (RTX): This medication is a chimeric mAb targeting cluster of differentiation 20 on B-lymphocytes, leading to suppressed humoral immunity and an increased risk of infections. Although immunosuppressive treatment is known to increase the risk of infection, safety data on infectious complications in RA patients following RTX infusion remain limited. In a report by Javelot et al. (2020), the authors present a case of a patient with severe RA who developed herpetic stomatitis, which was further complicated by MRONJ after RTX therapy [33].

Nivolumab: This medication is a programmed death-1 inhibitor that enhances antitumor immune responses and has demonstrated clinical efficacy in treating malignancies such as renal cell carcinoma, non-small cell lung cancer, and melanoma [34]. Ahdi et al. (2023) analyzed data from the Food and Drug Administration Adverse Event Reporting System database (2010-2021) and reported that several newly introduced medications, including nivolumab, have been associated with ONJ. Nivolumab, which enhances antitumor immune responses, is used in the treatment of malignancies such as non-small cell lung cancer, melanoma, and renal cell carcinoma [34].

Ipilimumab: This medication is a humanized mAb that targets cytotoxic T lymphocyte-associated antigen-4 (CTLA-4), a receptor found on activated and regulatory T cells. By blocking CTLA-4, T-cell activity is improved, thereby enhancing immune responses against tumors, particularly in cases of metastatic melanoma. Guida et al. (2021) described a case of a 58-year-old woman with BRAF-mutated metastatic melanoma who developed MRONJ following extraction of a mandibular third molar. The patient reported persistent oral pain, and among all medications administered, ipilimumab was the only agent that was linked to MRONJ. This case highlights the importance of careful dental monitoring in patients undergoing treatment with novel chemotherapy medications [35].

mTOR Inhibitors

The mTOR signaling pathway plays a crucial role in interpreting environmental signals to regulate homeostasis and cellular growth. Dysregulation of this pathway has been associated with a range of disorders, such as obesity, type 2 diabetes, cancer, and neurodegenerative diseases [47]. Additionally, mTOR inhibitors appear to exhibit AA effects by preventing the production of VEGF and PDGFs, further contributing to their role in disease progression [15].

Temsirolimus: This medication is a potent mTOR pathway inhibitor and an ester analog derivative of rapamycin specifically developed for intravenous use to overcome the limitations of rapamycin’s poor oral bioavailability and inconsistent absorption. It maintains strong mTOR inhibitory activity and has shown improved clinical efficacy, particularly in the treatment of advanced renal cell carcinoma [48]. However, like other targeted therapies, temsirolimus has been associated with MRONJ. This association underscores the importance of thorough patient monitoring and proactive dental care to mitigate potential complications [34].

Everolimus: This medication is an inhibitor of the mTOR and is approved for the treatment of estrogen receptor-positive (ER+) advanced breast cancer in postmenopausal women receiving exemestane. Activation of the mTOR pathway promotes cancer cell growth, proliferation, and angiogenesis, contributing to resistance against endocrine therapies. By targeting this pathway, everolimus plays a key role in limiting tumor development and progression [36]. A case report by Lee et al. (2016) describes a 66-year-old postmenopausal woman who developed MRONJ after a tooth extraction while undergoing combined therapy with exemestane and everolimus for ER+ advanced breast cancer. Despite no history of osteoporosis or BP use, the patient presented with a painful, non-healing extraction site in the mandible four months after the procedure. This case highlights a potential link between everolimus and MRONJ, emphasizing the need for caution when performing dental extractions in patients receiving mTOR inhibitors [36].

Cytotoxic Agents

Emerging evidence suggests that cytotoxic agents like methotrexate and azacitidine may also contribute to MRONJ development [37,49].

Methotrexate: This medication is a first-line treatment for RA and an immunosuppressive drug for organ rejection prevention. It is also used in high doses for chemotherapy in cancers like leukemia and lymphoma. However, it can cause serious side effects, including synovitis, interstitial pneumonia, liver damage, and infections, and on the other hand, it has been linked to ONJ [49]. A case report detailed two patients with long-standing arthritis treated with methotrexate who developed ONJ, despite no history of AR or AA medication use [38].

Azacitidine: This medication is a cytosine nucleoside analogue, which works as a demethylating DNA and an antimetabolite, which decreases cell division and growth. These actions may hinder soft tissue healing and bone remodeling, which could have an impact on recovery following tooth extractions [37]. Nicolatou-Galitis et al. (2016) report a case of a 64-year-old male with acute myeloid leukemia receiving azacitidine therapy who was diagnosed with MRONJ, marking the first such case linked to this medication [37].

Therefore, these reports highlight the need for clinicians to be vigilant for MRONJ in patients undergoing cytotoxic chemotherapy, even in the absence of traditional risk factors.

BRAF Inhibitors

BRAF inhibitors, such as dabrafenib and trametinib, which target the v-Raf murine sarcoma viral oncogene homolog B1 (BRAF), have been associated with MRONJ, representing an emerging category of non-AR agents linked to this condition. Recent reports have also implicated the immune checkpoint inhibitor nivolumab. As the use of immunotherapies and targeted therapies like BRAF inhibitors continues to expand in cancer treatment, the risk of MRONJ in oncology patients is expected to rise. These developments highlight the need for increased awareness and enhanced education regarding the potential oral complications of these therapies [13].

AXL Kinase Inhibitors

Bemcentinib (BGB324): This medication is a selective inhibitor of the AXL receptor tyrosine kinase. It impairs angiogenesis and alters macrophage polarization, leading to increased secretion of pro-inflammatory cytokines such as IL-1β and TNF-α, which contribute to inflammatory tissue degradation and bone resorption. Bemcentinib-induced inhibition of angiogenesis, together with the increased inflammatory response, could favor a rapid necrotic progression of periodontitis [39]. In a reported case by Bumm et al. (2020), an 81-year-old male with secondary acute myeloblastic leukemia treated with bemcentinib for two months developed MRONJ, presenting with exposed necrotic bone. Despite discontinuing the medication immediately after MRONJ manifestation, the condition persisted. These findings suggest that bemcentinib may disrupt bone healing and inflammatory pathways, thereby increasing MRONJ risk. Meticulous preventive dental care before and during AXL inhibitor therapy is strongly recommended to mitigate this complication [39].

Other Immunosuppressors

Medications used in MM treatment: These immunomodulatory drugs used in MM treatment, including bortezomib, thalidomide, and its analogs like lenalidomide, increase the risk of MRONJ, particularly when combined with BPs such as zoledronic acid [40]. A case report by Caggiano et al. (2023) described a 60-year-old woman with MM who developed multi-drug-related ONJ after receiving lenalidomide, with AA properties and a direct cytotoxic effect, along with dexamethasone, bortezomib, and zoledronic acid. This highlights the heightened risk of MRONJ in patients on these treatments [40].

Pomalidomide: This medication is a thalidomide analogue that exhibits antineoplastic, AA, and immunomodulatory properties. It is primarily used in combination with dexamethasone for the treatment of MM. Although definitive evidence is limited, hypotheses have emerged suggesting that pomalidomide may contribute to the development of MRONJ [34].

Discussion

Mechanisms Underpinning MRONJ

MRONJ is a multifactorial condition characterized by necrotic bone exposure in the maxillofacial region, primarily associated with AR and AA therapies [1]. It is traditionally associated with AR agents, notably BPs and denosumab. However, recent studies have highlighted the role of other medications with a broader spectrum of drugs, including AA agents, corticosteroids, and chemotherapeutic agents, in the development of MRONJ [50].

MRONJ is believed to be caused by a complicated interaction between altered immune responses, decreased vascularization, and poor bone remodeling. AR treatments, on the other hand, reduce bone turnover and cause microdamage by blocking osteoclast action [1]. Patients with innate or acquired immune dysfunction are at an increased risk of developing MRONJ. Those with immunocompromised states or medical conditions such as RA or diabetes face a heightened risk of MRONJ, regardless of exposure to AR agents. Moreover, certain immune-modulating medications are recognized for increasing susceptibility to infections and may work in combination with AR agents to promote the onset of MRONJ [28].

Although the exact pathophysiology of MRONJ remains unclear, several hypotheses have been proposed. These include suppressed bone turnover, chronic infection and inflammation, compromised immune function, soft tissue toxicity, and reduced blood vessel formation [4].

Risk Factors

Various systemic and local factors contribute to the development of MRONJ. Patients undergoing treatment with oral or intravenous BPs and denosumab are at significantly increased risk. Additionally, other medications such as angiogenesis inhibitors, TKIs, mAbs, mTOR inhibitors, and immunosuppressants have been identified as emerging contributors to MRONJ. Systemic conditions that increase susceptibility include chemotherapy for malignancies such as MM and cancers of the breast, prostate, lung, kidney, and colon, as well as corticosteroid medications, diabetes, smoking, and cardiovascular diseases like hypertension, hyperlipidemia, and angina. Other conditions, including osteoporosis, RA, Sjögren’s syndrome, sarcoidosis, hypocalcemia, hypoparathyroidism, osteomalacia, vitamin D deficiency, renal dialysis, anemia, Paget’s disease of bone, erythropoietin therapy, cyclophosphamide therapy, alcohol consumption, and obesity, have also been linked to a heightened risk [6]. Local factors, particularly dentoalveolar surgery like tooth extraction, periodontal disease, dental implants, ill-fitting dentures, poor oral hygiene, and anatomical features like tori or sharp ridges, also significantly contribute to MRONJ pathogenesis [6].

Prevention

Preventing MRONJ is of critical importance, as it significantly reduces patient morbidity and improves quality of life. The AAOMS position paper update (2022) emphasizes that optimizing oral health prior to initiating AR or AA therapy can lower the risk of MRONJ development. This includes conducting thorough dental evaluations and treating existing infections or other oral pathologies before therapy begins [1]. Supporting this approach, a 2024 study by Alblazi et al. demonstrated that patients who received comprehensive dental care before initiating AR or AA therapy had a significantly reduced incidence of MRONJ, highlighting the preventive benefit of pre-treatment dental interventions [51].

Ruggiero et al. (2022) further reported that several modifiable risk factors should be addressed to minimize MRONJ risk [52]. These include completing invasive dental procedures prior to AR/AA therapy initiation, using pre- and postoperative antibiotics and antiseptic rinses, maintaining good oral hygiene, and achieving primary closure of extraction sites. Maintaining strict oral hygiene, alongside broader systemic measures such as smoking cessation and diabetes management, is also advised. While no single strategy can completely eliminate MRONJ risk, applying these measures collectively is strongly recommended to reduce incidence and severity [1].

Diagnosis

The typical clinical features of MRONJ include exposed necrotic bone, localized infection, and associated pain. The AAOMS classifies MRONJ into stages to guide clinical management. Stage 0 encompasses patients with no visible bone exposure but who may exhibit nonspecific symptoms or radiographic abnormalities. Importantly, studies have shown that nearly half of these patients may advance to stage 1, which has led AAOMS to consider stage 0 as a potential early indicator of the condition [1].

In stage 1, patients present with exposed necrotic bone or a probing fistula but remain asymptomatic and show no signs of infection. Radiographic findings, when present, are typically limited to the alveolar bone. Stage 2 is defined by similar bone exposure accompanied by clinical symptoms such as pain and signs of local infection. In Stage 3, the disease progresses further, with infected necrotic bone and complications such as bone necrosis extended beyond the alveolar bone (for instance, to the inferior border of the mandible or the maxillary sinus), oral-nasal or oral-antral communication, pathologic fractures, or advanced osteolysis [1,52].

Treatment

The management of MRONJ is stage-dependent, aiming primarily to control infection, relieve pain, and prevent disease progression. Conservative approaches are often the first line of treatment in early stages, like stages 1 and 2, including the use of antimicrobial mouth rinses, systemic antibiotics, and regular monitoring to reduce symptoms and limit infection [1]. In order to facilitate tissue regeneration and restore functional integrity, surgical debridement of necrotic bone is often indicated when conservative therapy is insufficient, especially in Stage 3 or persistent instances. However, nonoperative treatments could be necessary for patients with Stage 2 or Stage 3 disorders who are not eligible candidates for surgery [1].

The AAOMS 2022 guidelines emphasize that treatment should be personalized, taking into account the extent of necrosis, patient comorbidities, and therapeutic objectives to optimize outcomes [1]. In support of this principle, the 2024 position statement by the Italian Societies of Oral Pathology and Medicine and the Italian Society of Maxillo-Facial Surgery recommends a multidisciplinary approach that includes dental, surgical, and medical input and recognizes the utility of both conservative and surgical interventions depending on disease severity [53]. They also highlight the potential role of drug holidays in selected patients, although the evidence remains inconclusive, and such decisions must be based on risk-benefit assessment [53].

Recent studies indicate that an increasing number of drugs have been associated with MRONJ in recent years, although the strength of evidence varies [50]. Given the significant public health implications of MRONJ, it is essential for healthcare providers to remain informed about all high-risk medications, current prevention strategies, and updated consent protocols [50].

Limitations

This review has several limitations that should be acknowledged. First, the inclusion of case reports, while valuable for identifying potential emerging associations between drugs and MRONJ, constitutes a low level of evidence and may be subject to publication bias. These individual reports often lack standardized diagnostic criteria, control groups, and long-term follow-up, which limits the generalizability of their findings. Additionally, the literature reviewed encompasses studies with heterogeneous methodologies, sample sizes, and outcome measures, making it difficult to draw direct comparisons or synthesize results comprehensively. Consequently, while this review offers a broad overview of the current evidence, further high-quality, prospective, and controlled studies are needed to validate suspected drug associations and inform clearer clinical guidelines.

## Conclusions

Although denosumab and BPs are well-known contributing factors to MRONJ, recent studies have identified additional medications potentially associated with MRONJ, including AA drugs, corticosteroids, immune modulators, chemotherapies, and polypharmacy. Therefore, clinicians should remain vigilant for MRONJ in patients receiving these medications, even in the absence of traditional risk factors. This review highlights the evolving understanding of MRONJ, expanding its etiological framework beyond the conventional medication classes to include novel pharmacological agents. Further research is needed to clarify the underlying mechanisms of MRONJ, improve risk assessment, and establish more targeted prevention and treatment strategies to reduce its complications.
